# Anemoside A3 Inhibits Macrophage M2-Like Polarization to Prevent Triple-Negative Breast Cancer Metastasis

**DOI:** 10.3390/molecules28041611

**Published:** 2023-02-07

**Authors:** Peng Liu, Yahui Liu, Lanying Chen, Zeping Fan, Yingying Luo, Yaru Cui

**Affiliations:** 1 National Pharmaceutical Engineering Center for Solid Preparation in Chinese Herbal Medicine, Jiangxi University of Chinese Medicine, Nanchang 330006, China; 2 Key Laboratory for Evaluation on Anti-Tumor Effect of Chinese Medicine by Strengthening Body Resistance to Eliminate Pathogenic Factors, Nanchang 330006, China

**Keywords:** M2 polarization, Anemoside A3, triple-negative breast cancer, metastasis, STAT3

## Abstract

Triple negative breast cancer (TNBC) exhibits the characteristics of strong metastatic ability and a high recurrence rate, and M2-type macrophages play an important role in this process. Previous research data suggested that Anemoside A3 (A3), a monomeric component of Pulsatilla Chinensis, could prevent and treat TNBC by converting M0 macrophages into M1 immunogen phenotypes. This study showed that A3 significantly restrained the lung metastases of 4 T1-Luc cells with bioluminescence imaging in vivo and Hematoxylin and Eosin (H&E) staining. Meanwhile, the percentage of M2-type macrophages (CD206+ labeled cells) in the lung tissues was evidently decreased through immunohistochemical assay. We further proved that A3 markedly prevented M2-type polarization induced by IL-4 in vitro, as illustrated by the down-regulated expression of the cell surface marker CD206 protein by FACS and Arg-1, and of the Fizz1 and Ym1 genes by RT-PCR in M2-type macrophages. Furthermore, the invasion and migration of 4 T1 cells, which was promoted by the conditioned medium from M2-type macrophages, could be suppressed by A3. Luminex assay demonstrated that A3 treatment resulted in a reduction of the levels of CCL2, VEGF, CCL7, and MMP-9 in conditioned medium. Additionally, the expression of phosphorylated-STAT3 protein was inhibited by A3, which resulted in the macrophage M2-type polarization arrest, while no significant difference in JAK2 phosphorylation was detected. SiRNA transfection experiments suggested that STAT3 might be the target of A3 inhibiting M2-type polarization of macrophages. In conclusion, these results indicate that A3 could attenuate the metastasis of TNBC by inhibiting the M2-type polarization of macrophages, which may be related to the STAT3 pathway.

## 1. Introduction

TNBC accounts for 15% of all breast cancers, with high metastasis and recurrence rates [1]. It has the worst prognosis and lowest survival rate among all breast cancer subtypes [2]. TNBC patients lack effective targeted therapies due to the absence of biomarkers [3,4]. Therefore, it is urgent to search for safe and effective anti-TNBC metastasis drugs.

Tumor-associated macrophages (TAMs) are derived from peripheral blood mononuclear cells infiltrating into solid tumor tissue. TAMs account for 50% of tumor stromal cells in breast cancer [5,6,7]. The prognosis of more than 80% of breast cancer patients is closely related to TAMs [8]. Macrophages can polarize into classical activated macrophages (M1-type macrophages) [9,10] and alternative activated macrophages (M2-type macrophages) [11]. TAMs are domesticated by the tumor microenvironment and are characterized by the M2-type macrophage phenotype, which plays an important role in the process of tumor metastasis [12,13]. Therefore, M2-type macrophages are considered one of the potential targets of anti-TNBC metastasis drugs [8].

Numerous studies have attempted to using natural compounds to alter the phenotypes of macrophages in tumorigenesis, thereby preventing tumor progression and metastasis [14]. Chinese medicine Pulsatilla chinensis has a wide range of pharmacological effects, including anti-tumor, anti-virus, anti-bacterial, immune-enhancing, etc., effects. Anemoside A3 (A3) is derived from Pulsatilla chinensis. Previous screening experiments on different saponins of Pulsatilla showed that A3 could significantly regulate the polarization of macrophages, polarizing M0 macrophages into classical activated macrophages (M1-type), and inhibiting alternative activated macrophages (M2-type) [15]. In addition, we demonstrated that A3 inhibits breast cancer tumor growth and angiogenesis by promoting M1-type macrophage polarization and secretion of the proinflammatory cytokine IL-12 [15]. Based on previous works, this paper researched the effect of A3 on TNBC metastasis by regulating the M2-type polarization of macrophages, to provide an experimental basis for the prevention and treatment of TNBC metastasis through traditional Chinese medicine. In this study, to mimic lung metastasis of TNBC, a mouse model of lung metastasis of 4 T1-Luc breast cancer cells was constructed to evaluate the effect of A3 on TNBC metastasis [16,17]. The mouse bone marrow derived macrophage (BMDM) was induced to construct the primary M2 polarization model of macrophages to investigate the effect and mechanism of A3 on M2-type polarization.

## 2. Results

### 2.1. A3 Suppresses Triple-Negative Breast Cancer Metastasis In Vivo by Inhibiting TAMs Polarization to M2-Type

**Figure 1:**
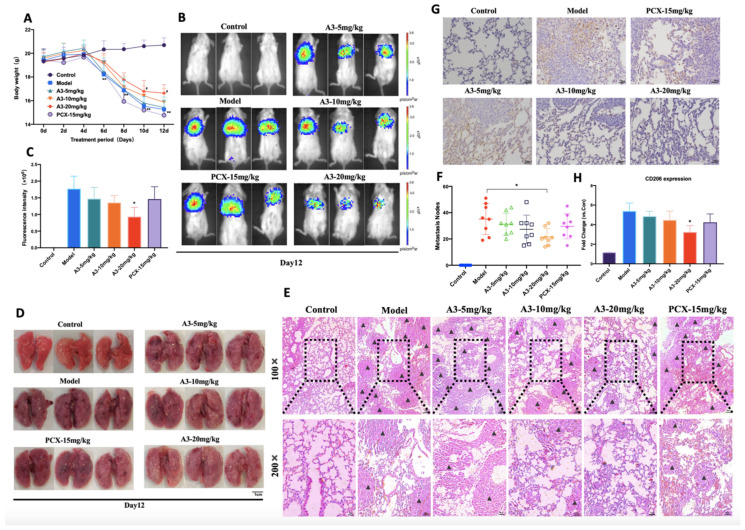
A3 reduced lung-metastasis in mice model of TNBC by preventing M2-type polarization. (**A**) Time-weight curve of mice injected with 4 T1-Luc cells in each group treated with different doses of A3 or Paclitaxel. (**B**,**C**) Representative images of the bioluminescence signal on the 12th day; Metastatic tumor growth in the lung was evaluated through measurement of the total fluorescence signal intensity at the metastatic site using Spectrum Living Image 4.0 software. (**D**) Lung tissues were isolated and photographed to count tumors on the 12th day. (**E**) Representative images of hematoxylin and eosin-stained sections of lungs from 4 T1 tumor-bearing mice are shown, and the black triangle represented metastatic nodules. (**F**) Quantification of metastatic nodules in the lungs after 4 T1 tumor cell challenge. (**G**) Representative immunohistochemical images of CD206 expression in lung tissues (×100 magnification, bar represent 25 μm). (**H**) Quantification of immunoreactive intensities of CD206. Negative control slide stained with isotype control IgG. All data shown represent means ± SEM (*n* = 6). Significant differences are indicated as * *p* < 0.05, ** *p* < 0.01, ns not significant.

The 4 T1-Luc breast cancer mice models were established to determine the effects of A3 in vivo. Apart from the normal group, the body weight of other mice began to decrease significantly on day 6 of administration (*p* < 0.01). The groups that were treated with A3 showed a slower tendency to weight loss, especially in the A3 high-dose group (*p* < 0.05), as shown in Table 1, Figure 1A. On the 12th day of modeling, significant reductions in fluorescence intensity were observed in tumor-bearing mice following A3 treatment (*p* < 0.05), as shown in Figure 1B,C.

To further investigate the effect of A3 on breast cancer metastasis in mice, the target organ lungs of tumor metastasis were dissected and photographed (Figure 1D). After HE staining, tumor metastasis nodes in the lungs were observed and counted, as shown in Figure 1E,F. The number of tumor metastasis nodes in the lungs of mice injected with A3 was significantly decreased (*p* < 0.05). Immunohistochemical results showed that, as compared to the model group, the expression of M2-type macrophage surface antigen CD206 in lung tissues was significantly reduced in the A3 groups (*p* < 0.05), as shown in Figure 1G,H.

### 2.2. A3 Attenuates IL-4-Induced M2-Type Polarization of BMDM Cells

**Figure 2:**
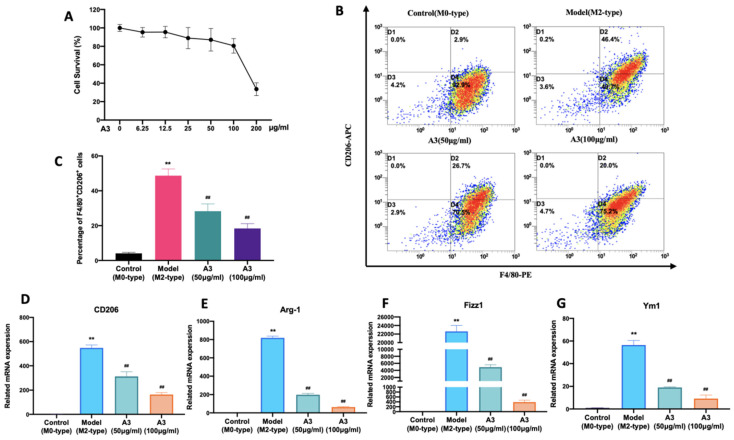
A3 inhibited M2-type polarization of macrophages induced by IL-4. (**A**) Effects of the different concentrations of A3 on the cell survival of BMDM using the CCK-8 assay. (**B**,**C**) Flow cytometric analysis and quantification of M2 surface marker CD206 expression in BMDM after A3 administration. (**D**–**G**) The mRNA expression of M2 marker genes in BMDM after A3 treatment determined by Quantitative Real-time PCR. All data shown represent means ± SEM (*n* = 3). Significant differences are indicated as ** *p* < 0.01 compared with Control group, **##** *p* < 0.01 compared with Model group.

We used the CCK-8 method to detect the effect of A3 on the survival rate of BMDM cells. Results shown in Figure 2A illustrate that A3 at the concentration range of 0–100 μg/mL had no significant effect on the survival rate of BMDM cells (*p* > 0.05). Therefore, we selected 100 μg/mL and 50 μg/mL as the high and low dose of the drug. Subsequently, flow cytometry was used to detect the effect of A3 on macrophage M2-type polarized surface antigen CD206. Flow cytometry results showed that the proportion of F4/80+CD206+ cells was significantly up-regulated in M2-type macrophages induced by IL-4 (*p* < 0.01). After the A3 treatment, the double positive ratio of F4/80+CD206+ BMDM cells decreased significantly (Figure 2B,C). RT-PCR was used to further explore the effects of A3 on the transcription of the signature genes of M2-type macrophages. The experimental results are shown in Figure 2D–G. The mRNA expressions of CD206, ARG-1, Fizz1 and Ym1 were significantly decreased after treatment with A3 (*p* < 0.01), compared with M2-type macrophages derived from BMDM cells.

### 2.3. Inhibition of 4 T1 Cell Migration Is Macrophage-Dependent

**Figure 3:**
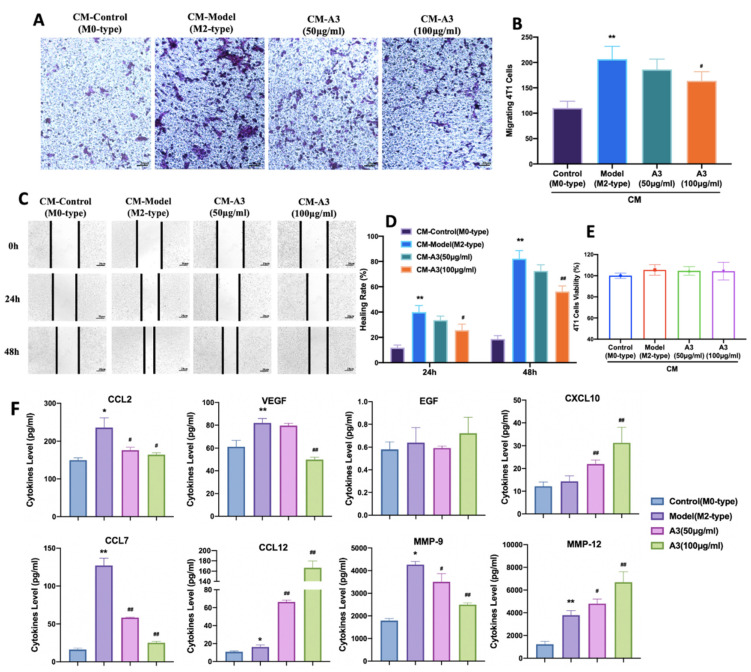
Inhibition of 4 T1 cell migration by BMDM treated with A3. (**A**,**B**) Cells were counted in ten random fields of view/well under an optical microscope (×100). (**C**,**D**) Images of the cells along the wound line were captured at 0 h, 12 h, and 24 h under an optical microscope with a ×10 objective lens. (**E**) Effects of BMDM-conditioned medium on the cell viability of 4 T1 cells using the CCK-8 assay. (**F**) The graphs represent the concentrations of CCL2, CCL7, CCL12, VEGF, EGF, CXCL10, MMP-9, and MMP-12 in conditioned medium determined by using multiplex Luminex. All data shown represent means ± SEM (*n* = 3). Significant differences are indicated as * *p* < 0.05, ** *p* < 0.01 compared with Control group, **#** *p* < 0.05, **##** *p* < 0.01 compared with Model group.

Previous experimental results showed that A3 has a significant inhibitory effect on the IL-4-induced M2-type polarization of macrophages. Next, we used the transwell cell migration experiment to investigate the effect of A3 on the migration of 4 T1 cells by inhibiting the M2-type polarization. The results showed that IL-4-induced BMDMs conditioned medium significantly increased the number of 4 T1 cells migrating to the bottom of the transwell chamber (*p* < 0.01). The conditioned medium treated with A3 significantly reduced the number of migrating 4 T1 cells (*p* < 0.05), as shown in Figure 3A,B. The results of the wound healing experiment showed that, following A3 intervention, the conditioned medium significantly reduced the wound healing rate of 4 T1 cells (*p* < 0.05), as shown in Figure 3C,D.

Meanwhile, we also investigated the effect of A3-treated conditioned medium of BMDMs on the proliferation ability of 4 T1 cells. The results showed that A3-treated conditioned medium had no significant effect on the proliferation of 4 T1 cells. These results indicate that A3 inhibits the migration of 4 T1 cells by inhibiting macrophage polarization rather than inhibiting 4 T1 cell proliferation, as shown in Figure 3E. Next, Luminex assay was used to detect the effects of A3 on cytokines and chemokines in BMDM-conditioned medium. The experimental data suggested that A3 treatment resulted in a reduction of the levels of CCL2, VEGF, CCL7, and MMP-9 and a rise in the levels of CCL12, CXCL10, and MMP-12 in conditioned medium, with no effects on EGF, as shown in Figure 3F.

### 2.4. A3 Inhibits M2-Type Polarization of Macrophages via STAT3 Dependent Pathway in BMDM Cells

**Figure 4:**
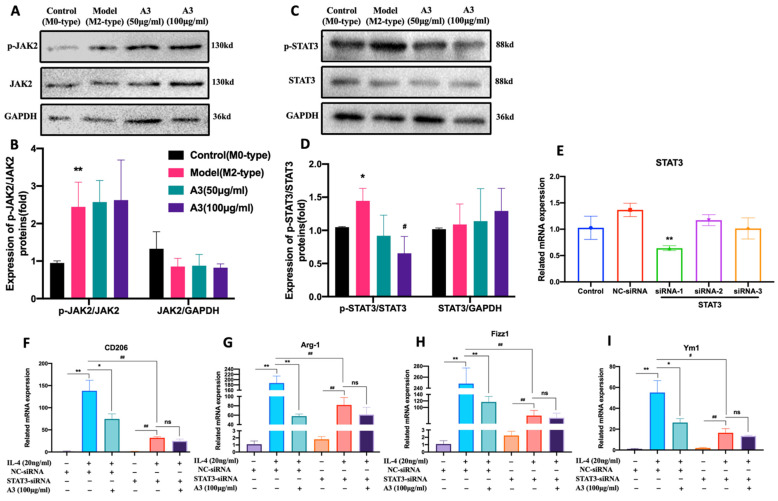
A3 impaired the M2 macrophages polarization in a STAT3-dependent manner. Western blotting analysis and quantification of protein expression levels of the phosphorylated proteins as well as the total proteins of Jak2 (**A**,**B**) and STAT3 (**C**,**D**) in BMDM with different doses of A3. (**E**) BMDM were treated with nontargeting control (NC) or STAT3-siRNA, and relative STAT3 mRNA expression was determined by Quantitative Real-time PCR. (**F**–**I**) The mRNA expression levels of M2 marker CD206, Arg-1, Fizz1, and Ym1 in BMDM were treated with NC siRNA or STAT3 siRNA and then stimulated with IL-4 alone or concomitantly stimulated with IL-4 plus A3 for 24 h; data are presented relative to GAPDH expression level and normalized to the mean expression level. All data shown represent means ± SEM (*n* = 3). Significant differences are indicated as * *p* < 0.05, ** *p* < 0.01 compared with Control group; **#** *p* < 0.05, **##** *p* < 0.01 compared with Model group.

To explore the mechanism of A3 inhibiting M2-type polarization of macrophages, we used the Western-blot method to detect the effects of A3 on the expression of JAK2 and STAT3 totals and phosphorylated proteins in BMDM cells. The results of the Western-blot showed that the levels of JAK2 and STAT3 protein phosphorylation in BMDM cells were significantly increased in the M2 model group induced by IL-4 (*p* < 0.05). The protein expression of p-STAT3 in the A3 treatment group was significantly decreased (*p* < 0.05), while the protein expression of p-JAK2 did not change significantly, as shown in Figure 4A–D. These results suggested that A3 may inhibit the M2-type polarization of macrophages by inhibiting the phosphorylation of STAT3 protein.

STAT3 siRNA was transfected into primary BMDM cells. RT-PCR results showed that the STAT3-SiRNA-1 was the sequence with the highest efficiency of STAT3 knockdown (*p* < 0.05) (Figure 4E). And the mRNA expression levels of CD206, Fizz1, Ym1, and Fizz1 after STAT3 knockdown were significantly down-regulated (*p* < 0.01). However, the expression of those genes was not altered in A3 treatment after STAT3 silencing, compared with the nontreatment group. Experimental results suggested that A3 inhibited M2-type polarization of macrophages by intervening targeted STAT3, as shown in Figure 4F–I.

## 3. Discussion

Tumor microenvironment has been recognized as a crucial target in cancer immunotherapies. M2-type macrophages play an important role in the process of tumor metastasis [18,19]. It has been illustrated that TAMs account for 50% of tumor stromal cells in breast cancer [5,6,7]. Therefore, suppressing M2-type polarization of TAMs may be a promising method for preventing TNBC metastasis. Natural products derived from traditional Chinese medicine have the characteristics of unique structures and amazing bioactivities which have been a rich source of drug leads and candidates [20]. The previous study by our team demonstrated that A3 could prevent breast cancer tumor growth and angiogenesis by regulating macrophage polarization toward the M1-type immunogen phenotype [15]. However, a comprehensive analysis of the regulation of M2-type macrophage polarization by A3 has yet to be performed. In the current study, we uncovered that A3 could attenuate the lung metastasis of TNBC by blocking M2 polarization of macrophages. This study provides a rationale for utilizing traditional Chinese medicine extracts in the immunotherapy of breast cancer metastasis.

Many clinical and experimental findings have shown that M2-type macrophages can remodel the microenvironment of tumor metastatic tissues at tumor metastatic sites, making them more conducive to tumor cell genesis and development [21,22,23,24]. The findings of this study showed that treatment with A3 in 4 T1-Luc breast cancer mice inhibited the distribution of M2 type macrophages (CD206+ labeled cells) in the lung tissue, consistent with the reduction of tumor metastasis nodes observed in these mice. This is in line with the results in vitro, where the up-regulation of anti-inflammatory M2 markers CD206, Arg-1, Fizz1, and Fizz1 induced by IL-4 were all reduced upon A3 treatment. Clinical research reveals the infiltration of M2-type TAMs is associated with treatment failure and poor prognosis in different cancers [25,26]. Thus, we have reason to believe in the potential anti-tumor effect of A3 by regulating macrophage polarization.

It is known that M2-type TAMs induce an immunosuppressive tumor microenvironment to promote dissemination of malignant cells in the early stages of tumor metastasis [27,28]. This study confirmed that the condition medium from A3 pre-treated BMDMs cells inhibited the invasion and migration of 4 T1 cells. What is more, Li H. et al. elucidated that M2 macrophages could promote cancer cell migration and up-regulate the expression of VEGF and MMPs for angiogenesis and invasion [29]. We analyzed the supernatant from M2-type macrophages by Luminex assay. The results demonstrated that the expression of CCL2, VEGF, CCL7, and MMP-9 was significantly decreased in A3 treatment. MMP-9, a member of the matrix metalloproteinases (MMPs) family, could promote tumor metastasis via directly degrading the extracellular matrix [30,31]. The CCL2/CCR2 axis has been shown to not only regulate the infiltration and activation of monocytes, T lymphocytes, and NK cells, but to directly mediate angiogenesis, promote tumor invasion, and metastasis [32,33]. Based on the effect of A3 on M2-type macrophage polarization, we believe A3-influenced relevant mediators CCL2 and MMP-9 released by macrophages to inhibit angiogenesis and cancer cell migration. Nevertheless, we also found that the expressions of CXCL10, CCL12, and MMP-12 were significantly increased in condition medium after A3 treatment. There are few reports about these cytokines in M2 macrophages. Therefore, the role of these soluble mediators in TNBC metastasis needs further explanation and more rigorous research.

It is well known that in tumor tissues, macrophages are mainly polarized to the M2 type with over expression of anti-inflammatory mediators, while the M1 type with over expression of pro-inflammatory mediators is obviously rare. In this study, we found that A3 significantly suppressed the M2 phenotype of macrophages and reduced the expression of anti-inflammatory mediators CCL2, MMP-9, CCL7, and VEGF. Meanwhile, in our previous results, A3 could promote M1 polarization of macrophages in tumor tissues and increase the expression of the pro-inflammatory mediators TNF- α, IL-12, IL-6, and IL-1β [15]. Therefore, we can assume that use of A3 could, to some extent, correct the balance between the pro-and anti-inflammatory mediators. This characteristic of A3 could greatly increase its potential druggability.

Increasing evidence has revealed that the signal transduction and activator of the transcription (STAT) family are closely related to macrophage polarizations [34,35]. Previous research has shown that the M1-type polarization of macrophages is induced by LPS and IFN-γ with activation of STAT1 and NF-κB [36]. Yao et al. found that Imatinib inhibited STAT6 phosphorylation and nuclear translocation, resulting in macrophage M2-like polarization arrest [19]. By activating STAT3, TAMs and small-cell lung cancers could jointly promote tumor progression [37]. It is well-known that STAT3 plays an important role in the process of IL-4 and IL-13 affecting M2-type polarization of macrophages [38,39]. Here we found that IL-4-induced STAT3 phosphorylation was diminished by A3 in macrophages. Previous study illustrated that the receptor-associated tyrosine kinase 2 (JAK2) is activated and phosphorylated after the recruitment of IL-4 to its receptor (containing IL-4 Rα and IL-2 Rγc) [40]. The activated JAK2 then phosphorylates three tyrosine residues on receptors, which provide docking sites for STAT3. Activated STAT3 forms a dimer and translocation to the nucleus after phosphorylation to further regulate downstream gene expression [41]. Thus, we analyzed the phosphorylation of JAK2 to explore the activation of IL-4 R. Our results showed that A3 did not affect IL-4 induced JAK2 phosphorylation, suggesting that IL-4 R were not involved in A3 inhibited STAT3 activation.

We have confirmed to some extent that A3 inhibits M2-type polarization by targeting STAT3 protein through siRNA technology. However, the role of A3 in STAT3 knockout mouse models, and whether the inhibition of A3 on lung metastasis of TNBC is dependent on macrophage-derived cytokines and chemokines, remains unclear. Thus, further experiments will be needed to clarify these issues. In addition, an increasing number of studies have found that macrophage polarization and cell metabolism are closely related. Among them, mitochondrial function, stability, and mtROS production all have the potential to influence the direction of macrophage polarization and then regulate the M1/M2 ratio of macrophages [42]. There are data showing that LPS stimulation leads to an elevated mitochondrial membrane potential (ΔΨm), which leads to accumulation of mtROS and promotion of IL-1β gene induction, and promotes the M1 polarization of Macrophages. Moreover, in IL-4-stimulated macrophages (M2 phenotype), Prostaglandin E2 (PGE2) modulates the expression of genes encoding the malate-aspartate shuttle (MAS) and reduces levels of MAS metabolites, leading to the decrease of ΔΨm [43]. Whether Anemoside A3 can inhibit macrophage M2 polarization by affecting the metabolic pathways of macrophages merits further exploration.

## 4. Materials and Methods

### 4.1. Compounds and Drugs

Anemoside A3(A3) (purity >98%) was purchased from Chengdu Push-biotechnology Co., Ltd. (Chengdu, China). Paclitaxel (PCX) was acquired from SL Pharm Co., Ltd. (Beijing, China).

### 4.2. Cell Lines and Cell Culture

The mouse fibroblast cell line (L929) was purchased from the Chinese Academy of Sciences Cell Bank and the mouse triple-negative breast cancer cell line 4 T1-Luciferase (4 T1-Luc) was purchased from TongPai Biotechnology Co., Ltd. L929. The 4 T1-Luc cells were cultured in RPMI 1640 medium supplemented with 10%FBS (Gibco, ThermoFisher, Waltham, MA, USA) and 1% penicillin-streptomycin (Solarbio, Beijing, China). All cells were incubated at 37 °C in an atmosphere of 5% CO_2_.

### 4.3. Animal Care

Female C57 BL/6 mice (6–8 weeks) and female Babl/c mice (6–8 weeks) were purchased from the SJA Laboratory Animal Co., Ltd. (Changsha, China), which were acclimated to the laboratory for 7 days before experimentation. All mice were maintained under SPF conditions in a controlled environment of 20–22 °C, with a 12 h light to 12 h dark cycle, at 50–70% humidity. Meanwhile, we provided food and water ad libitum for all mice. The animal study protocol was carried out in accordance with the recommendations and guidelines issued by the Animal Ethics Committee of the Jiangxi University of Traditional Chinese Medicine (Permit Number: JZLLSC2021–0015).

### 4.4. In Vivo Tumor Models

4 T1-Luc cells (200 μL, 5 × 106 cells) were injected into the tail vein of female Babl/c mice. After 24 h of inoculation, the mice respectively received 5 mg/kg, 10 mg/kg, 20 mg/kg A3 (once a day) or 15 mg/kg Paclitaxel (once every three days) by intraperitoneal injection. The weight of the mice was recorded the next day. The mice were humanely sacrificed at 12 d as the model control mice started showing signs of sickness.

### 4.5. In Vivo Bioluminescence Imaging

According to the manufacturer’s instructions, D-luciferin potassium salt (150 mg/kg, BioScience, Shanghai, China) was intraperitoneally injected 25 min prior to bioluminescence imaging. The mice were imaged using an in vivo imaging system (Xenogen IVIS Spectrum, Caliper, Princeton, NJ, USA). Excitation and emission filters were set at 620 and 670 nm.

### 4.6. He Staining and Immunohistochemical Analysis

Lung tissue was fixed in a 4% paraformaldehyde followed by paraffin embedding. The tissue wax blocks were cut into 5 μm slices by a tissue slicer. Following this, the sections were stained with hematoxylin and eosin (H&E). The 5 μm-thick tumor tissue slides were treated with 3.0% hydrogen peroxide and closed with sheep serum for immunohistochemical analysis. They were incubated with the CD206 primary antibody (1:400 dilution, Abcam, Cambridge, MA, USA) at 4 °C overnight, followed by incubation with horseradish peroxidase (HRP)-conjugated secondary antibody (goat anti-mouse IgG, CWBIO, Taizhou, China) at room temperature and detection with diaminobenzidine with hematoxylin as a reverse stain. Photography was performed using the LEICA DM2500 Optical Microscope.

### 4.7. Production of L929 Cell-Conditioned Medium (LCCM) and BMDM Differentiation

The supernatant of L929 cells cultured in RPMI 1640 medium for 5 days was collected, which was filtered under aseptic condition before aliquoting into 50 mL centrifuge tubes and being stored at −20 °C. Bone marrow-derived cells were aseptically harvested from female C57 BL/6 mice by flushing the femurs and tibias of euthanized mice. BMDM cells were induced in an RPMI 1640 medium supplemented with 10%FBS, 1% penicillin–streptomycin and 20% LCCM for 6 days.

### 4.8. Cell Viability

According to the manufacturer’s protocol, cell viability assay was determined on the indicated days using the Cell-Counting Kit-8 (CCK-8; Dojindo, Kumamoto, Japan).

### 4.9. Flow Cytometry

BMDM were incubated with 0.1 µg fluorescent dye-labeled monoclonal antibodies F4/80 (PE, Biolegend, San Diego, CA, USA) and CD206 (APC, Biolegend, San Diego, CA, USA) at room temperature for 30 min. Finally, cells were washed, resuspended in FACS buffer, and analyzed by flow cytometry (Gallios, Beckman Coulter, Brea, CA, USA). The manufacturer’s recommended isotype controls were used as negative control.

### 4.10. Quantitative RT-PCR

According to the manufacturers’ instructions, total RNA from the macrophages was extracted with TRIzol (Invitrogen, Carlsbad, CA, USA). The Real-time PCR reactions were quantified by fast RT-PCR System (ABI 7500, Applied Biosystem, Foster, CA, USA). Amplification reaction assays were performed using SYBR Green PCR Master Mix (Applied Biosystem, Foster, CA, USA) and primers (100 nM, Genscript, Beijing, China), with β-actin as the internal control. The mouse primer sequences used in this study were as shown in Table 2.

### 4.11. Production of BMDM-Conditioned Medium

BMDM cells were stimulated with medium (negative control-M0), IL-4 (20 ng/mL, model-M2) or IL-4 plus A3 for 24 h. The supernatant of cells was then carefully removed and replaced with fresh RPMI 1640 medium for a subsequent 24 h. The supernatant collection was then sterile filtered as BMDM-conditioned medium and stored at −80 °C.

### 4.12. Transwell Matrix Penetration and Wound Healing Assay

4 T1 cells were seeded into the upper chamber of polycarbonate transwell filters (Corning Inc., Corning, NY, USA). After incubation for 12 h, the cells were fixed in 25% precooled ethanol and stained with crystal violet, which had invaded to the bottom surface of the membrane. Cells were counted in 10 random fields of view/well under an optical microscope (×100). The number of 4 T1 cells invading to the bottom in each field was calculated by using Image-Pro-Plus.

4 T1 cells were cultured in 6-well plates until a monolayer of cells was formed. Next, a straight linear wound was created in the middle of the cell monolayer using a sterile pipette tip. Images of the cells along the wound line were captured at 0 h, 12 h, and 24 h after wounding under an optical microscope with a ×10 objective lens. The percentage of the area closed in 12 h and 24 h was measured using Image J. The ratio of the wound closing area to the total area of the initial gap was regarded as the wound closing rate.

### 4.13. Quantification of Cytokines and Chemokines

Quantification of cytokines and chemokines in previously collected BMDM-conditioned medium was performed using the Luminex™ x-MAP technology (Luminex-200, ThermoFisher, Austin, TX, USA) according to the manufacturer’s instructions and expressed in picogram/milliliter of medium.

### 4.14. Western Blot Analysis

WB analysis was performed as previously described [15]. Anti-bodies included STAT3 (68153, Abcam, Cambridge, MA, USA); p-STAT3 (76315, Abcam, Cambridge, MA, USA); JAK2 (108596, Abcam, Cambridge, MA, USA); p-JAK2 (32101, Abcam, Cambridge, MA, USA); and GAPDH (10494–1-AP, Proteintech, Wuhan, China). Proteins were visualized using the ECL Western Blotting Detection System (Chemi Doc XRS, Bio-Rad, Hercules, CA, USA) and quantified using Image J software (NIH, Bethesda, MD, USA).

### 4.15. Small Interfering RNA (siRNA) Transfections

The differentiated BMDM were transfected separately with siRNA-STAT3, siRNA-scramble (negative control-NC) (30 nM, OriGene Technologies, Inc., Rockville, MD, USA). Transfections of siRNA into the macrophages were performed according to the protocol provided by the manufacturer’s recommendations (Lipo3000, ThermoFisher, Austin, TX, USA).

### 4.16. Statistical Analyses

Data are the mean ± SD. The significance of results was determined based on one-way analysis of variance using (SPSS v. 18.0 software, IBM, Armonk, NY, USA). A result of *p* < 0.05 was considered significant. All the experiments were repeated three times.

## 5. Conclusions

**Figure 5:**
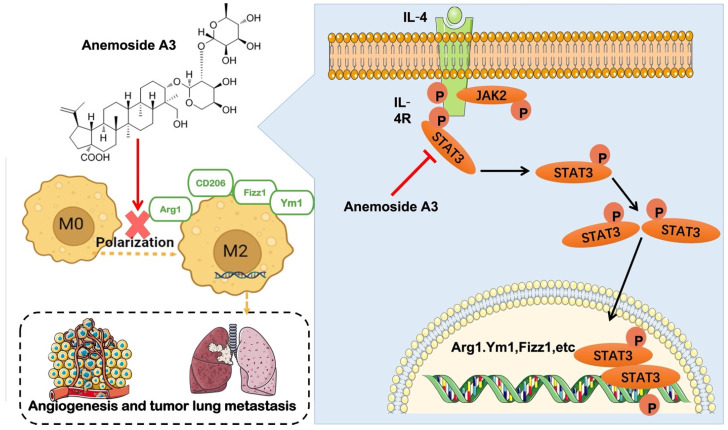
Diagram of how M2-type polarization of macrophages could be affected by A3 to reduce lung metastasis of breast cancer. A3 inhibits M2-type polarization by reducing STAT3 protein phosphorylation, leading to the suppression of M2-type macrophage induced tumor metastasis.

The results of the present study demonstrated that A3 could prevent lung metastasis of TNBC by inhibiting M2-type polarization of macrophages. The underlying mechanism of this process possibly includes suppression of STAT3 phosphorylated protein expression and interference with the STAT3 pathway, as shown in Figure 5. Although it has been proved that Pulsatilla Chinensis plays an immunomodulatory and anti-tumor role, the regulation of M2-type polarization has not been reported on. This study was the first to reveal the inhibitory effect on M2-type polarization of A3, a monomeric component of the plant, as well as its capacity to prevent metastasis of breast cancer. In summary, these results indicate that Anemoside A3 may be a promising natural compound for further research on the development of novel cancer therapeutics, particularly metastatic TNBC.

## Figures and Tables

**Table 1:** Effects of Anemoside A3 at different doses on weight in 4 T1 breast cancer mice ($\bar{x}$ ± *s*, *n* = 6).

Groups	Body Weight (g)						
	0 d	2 d	4 d	6 d	8 d	10 d	12 d
Control	19.33 ± 0.94	19.58 ± 0.92	19.84 ± 0.55	20.33 ± 0.73	20.35 ± 0.48	20.60 ± 0.52	20.69 ± 0.62
Model	19.41 ± 0.39	19.86 ± 0.43	19.93 ± 0.81	18.21 ± 1.14 **	16.88 ± 0.79 **	15.53 ± 0.80 **	15.27 ± 0.77 **
A3–5 mg/kg	19.71 ± 0.56	20.14 ± 0.72	20.43 ± 0.69	18.41 ± 1.19	16.94 ± 0.98	15.80 ± 0.53	15.43 ± 0.88
A3–10 mg/kg	19.60 ± 0.47	19.99 ± 0.83	20.24 ± 0.40	19.10 ± 0.75	17.14 ± 0.94	16.41 ± 0.83	15.87 ± 1.14
A3–20 mg/kg	19.28 ± 0.73	19.54 ± 0.45	19.99 ± 0.51	19.30 ± 0.69	17.76 ± 0.58	16.79 ± 0.92 **#**	16.64 ± 0.72 **#**
PCX-15 mg/kg	19.43 ± 0.93	19.22 ± 0.30	19.67 ± 0.95	18.30 ± 1.08	15.93 ± 0.82	15.20 ± 0.55	14.77 ± 0.54

Significant differences are indicated as ** *p* < 0.01 compared with Control group; **#** *p* < 0.05 compared with Model group.

**Table 2:** Primers and oligonucleotides used for RT-PCR.

Gene	Sense Strand (5′-3′)	Antisense Strand (3′-5′)
CD206	AGGGACCTGGATGGATGACA	TGTACCGCACCCTCCATCTA
Arg-1	AACACGGCAGTGGCTTTAAC	GTCAGTCCCTGGCTTATGGTT
Fizz1	CCCTGCTGGGATGACTGCTA	TGCAAGTATCTCCACTCTGGATCT
Ym1	GGCGCTGTCATCGATTTCTT	ATAGAGTCGCCACCCTGATG
β-actin	GGTCATCACTATGGCAACG	ACGGATGTCAACGTCACACT

## Data Availability

Not applicable.

## Sample Availability

Samples of the compounds are available from the authors.
